# Evaluation of markers of beige adipocytes in white adipose tissue of the mouse

**DOI:** 10.1186/s12986-016-0081-2

**Published:** 2016-03-18

**Authors:** Rolando A. Garcia, James N. Roemmich, Kate J. Claycombe

**Affiliations:** USDA-ARS, Grand Forks Human Nutrition Research Center, 2420 2nd Ave. N, Grand Forks, ND 58203 USA

**Keywords:** Beige, Brite, Adipocyte marker, Subcutaneous adipose tissue, UCP1, FGF21

## Abstract

**Background:**

There is a growing interest in exploiting the induction of beige or “brite” (brown in white) adipocytes (beigeing) to combat obesity and its comorbidities. However, there is some uncertainty regarding the best markers to evaluate the occurrence or magnitude of beigeing in white adipose tissue in the mouse model.

**Methods:**

We evaluated the transcript expression of several thermoregulatory genes and proposed beige markers employing cell culture, whole white adipose tissue, and the adipocyte and stromal vascular fractions.

**Results:**

Most beige markers tested with the exception of TMEM26 can discriminate white from beige adipocytes in culture. Markers FGF21, P2RX5, PAT2, or CAR4 can successfully mark beigeing in whole tissue of younger mice, or in the adipocyte subfraction of older mice. However, markers for the thermoregulatory genes UCP1, CIDEA, and Cox8b displayed the greatest dynamic range and were consistently elevated *in vitro*, *in vivo*, and in the adipocyte fraction by treatments that induce beige adipogenesis.

**Conclusions:**

While most putative beige markers are clearly expressed in beige adipocytes *in vitro*, *in vivo* the small dynamic range of most of these markers, the strength of the beigeing stimulus, and the age of the mice may limit their utility, although this limitation may be overcome by specifically evaluating these markers in the adipocyte fraction. Thermoregulatory markers like UCP1, CIDEA, or Cox8b represent the best options to evaluate the beigeing of white adipose tissue *in vivo*.

**Electronic supplementary material:**

The online version of this article (doi:10.1186/s12986-016-0081-2) contains supplementary material, which is available to authorized users.

## Background

Beige or “brite” (brown in white) adipocytes arise in white adipose tissue (WAT) in response to various stimuli including cold exposure and exercise. Unlike white adipocytes, which tend to have a single fat droplet (unilocular morphology) and low mitochondrial density, beige adipocytes have several small fat droplets (multilocular morphology) and a greater mitochondrial density [1]. Beige adipocytes as well as their counterparts in brown adipose tissue express the protein UCP1, which uncouples respiration from energy production resulting in the generation of heat [1]. UCP1 knockout mice reared at thermoneutrality develop obesity [2]. Increased activity of beige or brown adipocytes in transgenic animal models results in resistance to obesity [3]. When the amount of brown adipose tissue is greatly reduced in mice by genetic means, the induction of beige adipocytes in white adipose tissue can restore the thermogenic response to cold and prevent the mice from becoming more susceptible to weight gain in response to a high-fat diet [4]. Mice lacking functional beige adipocytes are more prone to developing obesity, insulin resistance, and hepatic steatosis when fed a high-fat diet [5]. In addition, transplantation of subcutaneous adipose tissue where beiging was induced by exercise into mice fed a high-fat diet restored glucose tolerance and insulin sensitivity [6]. Thus, there is interest in the possibility of exploiting the induction of beige cells to address maintenance of a healthy body weight and obesity-related diseases.

Studies seeking to increase beige adipogenesis (beigeing) or determine how it is affected by environmental treatments such as high-fat diets require markers of beige adipocytes. The expression of several thermoregulatory genes such as Uncoupling Protein-1 (UCP1), Cell Death-Inducing DFFA-Like Effector A (CIDEA) or Cytochrome C Oxidase Subunit VIIIb (Cox8b) has been employed to evaluate the induction of beige adipocytes in white adipose tissue (WAT). In addition, transcripts of several markers (e.g., TBX1, TMEM26, CD137, FGF21, P2RX5, PAT2, CAR4, and CITED1) have been identified that appear to be specific for beige adipocytes [7–9]. However, it is not yet clear whether employing these beige markers to gauge the extent of beigeing in WAT represents an improvement over the evaluation of the transcripts of thermoregulatory genes. There are also discrepancies as to the specificity of some of these markers to identify beigeing. Wu and coworkers [7] employing microarray screens of immortalized cells lines derived from murine subcutaneous fat identified TBX1, TMEM26, and CD137 as markers for beige adipocytes. However, Ussar and coworkers [9] did not detect the transcripts for TBX1 or TMEM26 to be changed in response to cold adaptation or treatment with a β3 adrenergic agonist [9], while Rosenwald and coworkers found that of these 3 markers only TBX1 was enriched in beige adipocytes and increased by cold stimulation [10]. A recent study [11] performed a comprehensive examination of several proposed beige adipocyte markers, however the focus of that study was to validate markers for identification of adipose tissues such as brown (interscapular), brite (inguinal), and white (epididymal) and not markers of beige adipocytes.

The aim of the present study was to determine differences in expression of classical thermoregulatory genes and putative beige markers under conditions that induce beigeing. The objective was to identify those markers that in general may be most useful to researchers employing mouse models to evaluate changes in beigeing in WAT in response to experimental treatments, or to researchers seeking to differentiate between white and beige adipocytes in cultured cells.

## Methods

### Animals

All animal protocols were approved by the Institutional Animal Care and Use Committee of the US Department of Agriculture, Agricultural Research Service, Grand Forks Human Nutrition Research Center. Male C57BL/6 mice were purchased from Harlan (Madison, WI), housed in a pathogen-free room on a 12:12 h light–dark cycle with unlimited access to rodent chow (#5015; LabDiet, St. Louis, MO) and water, and maintained at 22 °C unless otherwise specified. We employed the temperature of 22 °C for housing of the mice because it is within the range of the most common room temperatures employed in experiments. We used C57BL/6 mice because they are a strain of mice prone to obesity that is often used in this type of research. To induce beigeing in WAT, groups of mice (*n* = 5–6) were placed at 5 °C for one week in an environmental chamber. We employed cold exposure because it is the classical paradigm for beige adipocyte induction [1]. For the harvesting of tissues mice were euthanized by either CO2 inhalation or cervical dislocation. Tissue samples were frozen and stored at −80 °C until analysis.

### Cell culture

Inguinal adipose tissue from male C57Bl/6 mice was minced and digested in 0.5 % type I collagenase (ThermoFisher Scientific, Waltham, MA) in Hank’s balanced salt solution (HBSS) for 1 hr. at 37 °C. The digests were filtered through a 100 μm filter and centrifuged at 200 g for 7 min at 4 °C. After removal of the supernatant, the stromal vascular fraction (SVF) cell pellet was subjected to hypotonic RBC lysis buffer to remove erythrocytes. The cells were then cultured on a T25 in 5 mM glucose DMEM with 10 % FBS and 50 U penicillin and 50 μg of streptomycin/ml and subcultured to a 24 well plate. After cells reached confluence they were induced to differentiate into white adipocytes employing the same culture medium containing 0.5 mM Isobutylmethylxanthine (IBMX), 1 uM dexamethasone, 850 nM insulin, and 17 mM glucose (modified from Wu et al., 2012 [7]). For differentiation into beige adipocytes, 1 uM rosiglitazone and 1 nM T3 were also included. We employed rosiglitazone as an inducer of beiging because it is the most often used method for induction of beige adipocytes *in vitro*. After 4 days in differentiation medium, the white adipocytes were cultured for an additional 4 days in maintenance medium containing only insulin, while beige cells were cultured in the same medium containing 1 uM rosiglitazone and 1 nM T3 for an additional 2 days, and then in medium with only insulin for the last 2 days. About 90-95 % of the cells in the plates differentiated into adipocytes. At the end of the eighth day, cells were treated with 1 uM isoproterenol (Iso) for 6 hrs. We employed adrenergic stimulation in our cultures because the induction of beige adipocytes in white adipose tissue by stimuli like cold exposure is mediated by an increase in adrenergic stimulation, and we wished to examine its effects on gene expression markers in both beige and white adipocytes in culture.

### Separation of the stromal vascular fraction from the adipocyte fraction

The collagenase digests of inguinal adipose tissue were filtered through a 200 μm filter and diluted ½ with HBSS containing 10 % FBS to stop collagenase activity. After centrifugation (200 g, 7 min, 4 °C) the supernatant containing the floating adipocytes was decanted and centrifuged again before removing the infranatant to obtain the adipocyte fraction. To the cell pellet constituting the SVF 5 ml of HBSS were added and after centrifugation the supernantant was removed. Both the adipocyte and stromal vascular fractions were lyzed with QIAazol lysis reagent (Qiagen, Valencia, CA).

### qPCR

Frozen tissue or cells were homogenized in QIAzol lysis reagent and total RNA was isolated from tissue or cell lysates employing the RNeasy Lipid Tissue Mini Kit (Qiagen, Valencia, CA). RNA was converted to cDNA employing the High Capacity cDNA Reverse Trancription kit from Applied Biosystems (Foster City, CA). The transcripts of the thermoregulatory and beige genes evaluated in this study and their function are listed in Table 1. Additional transcripts for the following genes were evaluated: Solute Carrier Family 7 Member 10 (Slc7a10/ASC1 neutral amino acid transporter, y + system), WAP four-disulfide core domain 21 (Wfdc21/Wdnm1-like), and Serine (or Cysteine) Peptidase Inhibitor, Clade A, Member 3 k (Serpina3k). The beige markers evaluated in this study were selected either because they are among the markers most often used in mice, they have a confirmed role in the process of beigeing (FGF21), or the genes code for cell surface proteins that may prove useful in targeting beige or brown adipocytes for diagnostic or therapeutic purposes (PAT2 and P2RX5). Transcripts were evaluated employing a 5’ nuclease qPCR assay using the Faststart Universal Probe Master (Rox) from Roche (Indianapolis, IN) and 18S-rRNA as an endogenous control. Primer sets and hydrolysis probes (Table 2) were obtained from Integrated DNA Technologies (Coralville, IA). Ct values were determined using the ABI Prism 7500 PCR system (Applied Biosystems, Foster City, CA). Relative amplification was determined by the ∆∆Ct method.

**Table 1:** List or thermoregulatory or beige markers employed in this study and their function

Thermoregulatory or Beige marker	Function
UCP1 (Uncoupling protein 1)	Uncouples respiration from energy production
CIDEA (Cell Death-Inducing DFFA-Like Effector A)	Modulates uncoupling action of UCP1
Cox8b (Cytochrome C Oxidase Subunit VIIIb)	Subunit of oxidase involved in respiratory electron transport
FGF21 (Fibroblast Growth Factor 21)	Modulates glucose, lipid, and energy homeostasis: induces beigeing in WAT
Car4 (Carbonic Anhydrase 4)	Acid–base balance (bicarbonate/CO2 equilibrium)
P2RX5 (Purinergic Receptor P2X, Ligand-Gated Ion Channel 5)	Purinergic Signaling
PAT-2 (Proton-Coupled Amino Acid Transporter-2)	Amino acid transport
CITED1 (Cbp/p300-Interacting Transactivator with Glu/Asp-rich Carboxy-Terminal Domain 1)	Transcriptional coactivator
CD137/Tnfrs9 (Tumor Necrosis Factor Receptor Superfamily, Member 9)	Inflammatory reactions
TBX1 (T-Box 1)	Transcription factor (proliferation and differentiation of cells during organogenesis)
TMEM26 (Transmembrane Protein 26)	Unknown

**Table 2:** List of primer/probe sets

Gene	Forward primer	Reverse primer	Probe
UCP1	GCATTCAGAGGCAAATCAGC	GCCACACCTCCAGTCATTAAG	56-FAM/ACTGAGTCG/Zen/TAGA GGCCAATCCTGA/3IABkFQ
CIDEA	CATACATGCTCCGAGTACTGG	CATCCCACAGCCTATAACAGAG	56-FAM/TAACCAGGG/Zen/CACA GCTACAGAGG/3IABkFQ
Cox8b	AGCCAAAACTCCCACTTCC	TCTCAGGGATGTGCAACTTC	56-FAM/AGATCCCCA/Zen/C AGCCTGCTCC/3IABkFQ
Serpina3k	GCCAAAGTCAATAACCCCAAG	CTTTGCAACAGCCAATCAGAG	56-FAM/AGCCCTGGA/Zen/C AGAATCATGAGAAC/3IABkFQ
Wdnm1-like	GCCAGAGGAACAATGTGTCAG	GTAATCTCCATACATGGCCTCC	56-FAM/TGGTCACAG/Zen/AGCT CCAAACAGACAC/3IABkFQ
ASC1	TCTTCATTTCCATCCCACTGG	ATGACCCACGAAAAGTAGCC	56-FAM/ATGTCGCCT/Zen/ACTT CACTGCCATGT/3IABkFQ
FGF21	CAAATCCTGGGTGTCAAAGC	CATGGGCTTCAGACTGGTAC	56-FAM/CTCCATCTG/Zen/GCTG TTGGCAAAGAAA/3IABkFQ
P2RX5	TGATAGTTAATGGCAAGGCGG	TTGTCTCGGTAAAACTCGCTC	56-FAM/CCAGGTCAC/Zen/A GAAGAAAGCCCCA/3IABkFQ
PAT2	AGCCACCCCTCTCAATCT	TGCCTTTGACCAGATGAACC	56-FAM/CCAGGATCC/Zen/C AGTCCAGCGAATG/3IABkFQ/
CITED1	ATTTATCGGACTTCTGCCCAG	TTGCGATCCTTCACTCCAAG	56-FAM/AGGCCTCGA/Zen/CATA GTTGGCATTTCA/3IABkFQ
CAR4	CAGAGCACAGTATTGATGGGAG	CTTGTTCACCTTGTCTCCTACC	56-FAM/TTGTCCTTC/Zen/GAGT CCTCCTTGCTAGA/3IABkFQ
TBX1	TGGGACGAGTTCAATCAGC	TGTCATCTACGGGCACAAAG	56-FAM/TCCCCACGT/Zen/TCCA AGTGAAGCTTTT/3IABkFQ
CD137	CCTGTGATAACTGTCAGCCTG	TCTTGAACCTGAAATAGCCTGC	56-FAM/TGCCCTCCA/Zen/AGTA CCTTCTCCAG/3IABkFQ
TMEM26	GCACCATCACTAGAGACCAAC	ACAAGAATGCCAGAGACCAG	56-FAM/CGCCGTCCC/Zen/C ACAAACATCAGA/3IABkFQ
PDGFR-α	GTTGCCTTACGACTCCAGATG	TCACAGCCACCTTCATTACAG	56-FAM/TCCCAAAAT/Zen/CCGA CCAAGCACGA/3IABkFQ

### Western blot

White adipose tissue was homogenized in lysis buffer (50 mM HEPES, PH 7.4, 150 mM NaCl, 1 % Triton X-100, 5 mM EGTA) and homogenates were spun at 20,000 g. Supernatants were extracted with 4 volumes of cold acetone and the precipitated protein was resuspended in 10 % SDS, made up to a final SDS concentration of 2 % with water, and spun at 12,000 g for 2 min to obtain the final supernatant. Protein was determined by the BCA protein assay (Thermo Fisher, Rockford, IL). Proteins were separated in 10 % Bis-Tris NuPAGE gels and transferred to PVDF membranes employing the iBlot2 (Thermo Fisher, Rockford, IL). Membranes were blocked and incubated with primary and secondary antibodies in Odyssey blocking buffer (Li-Cor, Lincoln, NE). Primary antibodies were: anti-FGF21, AF3057, and anti-CD137, AF937 (R&D Systems, Minneapolis, MN), anti-P2RX5, sc-398682 (Santa Cruz Biotechnology, Dallas, TX), anti-beta-actin, sc-47778 (Santa Cruz, Dallas, TX), and anti-UCP1, ab10983, (abcam, Cambridge, MA). Dye-conjugated secondary antibodies were from Li-Cor (Lincoln, NE). The bands were detected by immunofluorescence.

### Statistics

Statistical analyses were carried out with the SIGMA Plot 13.0 software. Differences between two groups where tested using two-tailed t-tests. Differences between more than 2 groups were tested using two way ANOVAs followed by multiple comparisons using the Holm-Sidak method. If the normality and/or equal variance assumptions of the tests were not met, the data was transformed prior to analysis.

### Criteria for validation of beige adipocyte markers

We defined the following criteria for a putative marker to be a valid beige adipocyte marker: *In vitro*, the marker must be expressed in beige adipocytes at statistically significant greater levels than white adipocytes. The marker must be statistically significantly increased in subcutaneous white adipose tissue in response to stimuli that give rise to beige adipocytes and have a dynamic range of at least two fold. The marker should be expressed at statistically significantly greater levels in the adipocyte fraction compared to the SVF, and must be statistically significantly increased in the adipocyte fraction in response to stimuli that give rise to beige adipocytes.

## Results

### Beige adipocyte marker gene expression differences after white and beige adipocyte differentiation

**Fig. 1:**
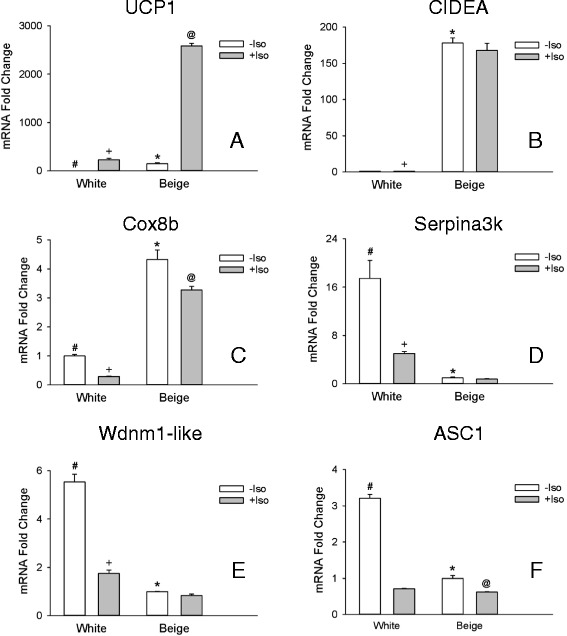
Thermoregulatory genes and white adipocyte markers *in vitro*. The transcripts for UCP1 **a**, CIDEA **b**, Cox8b **c**, Serpina3k **d**, Wdnm1-like **e**, and ASC1 (**f**) were evaluated in white and beige adipocytes in culture treated with isoproterenol (+ Iso) or without (− Iso) for 6 hrs. before harvesting the cells. Thermoregulatory gene transcripts were normalized to white –Iso, whereas white adipocyte gene transcripts were normalized to beige – Iso. Values are means ± SEM (*n* = 3). (*) different from white – Iso, (#) different from white + Iso, (+) different from beige + Iso, (@) different from beige – Iso, *p* ≤ 0.05, two way ANOVA followed by multiple comparisons using the Holm-Sidak method

**Fig. 2:**
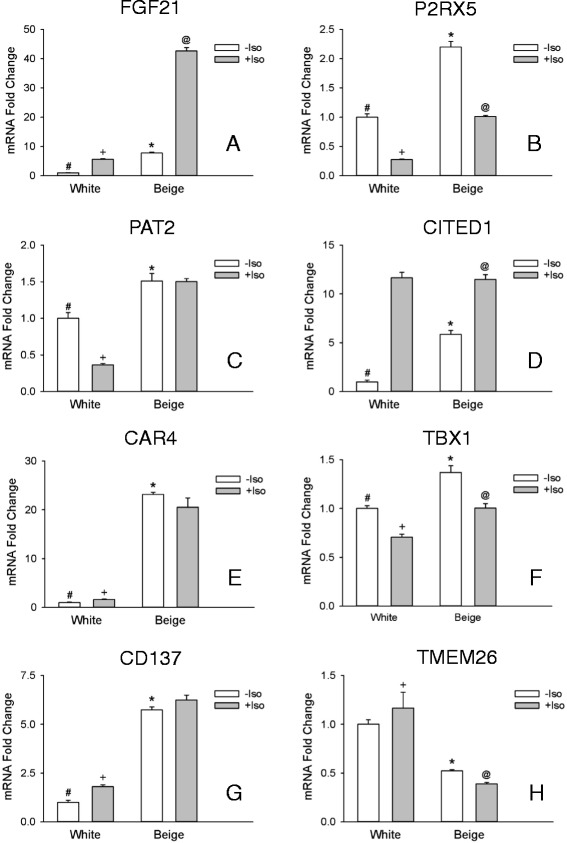
Markers of beige adipocytes *in vitro*. The transcripts for FGF21 **a**, P2RX5 **b**, PAT2 **c**, CITED1 **d**, CAR4 **e**, TBX1 **f**, CD137 **g**, and TMEM26 (**h**) were evaluated in white and beige adipocytes in culture treated with isoproterenol (+ Iso) or without (− Iso) for 6 hrs. before harvesting the cells. Transcripts were normalized to white – Iso. Values are means ± SEM (*n* = 3). (*) different from white – Iso, (#) different from white + Iso, (+) different from beige + Iso, (@) different from beige – Iso, *p* ≤ 0.05, two way ANOVA followed by multiple comparisons using the Holm-Sidak method

Expression of the thermoregulatory genes UCP1, CIDEA, and Cox8b was elevated 147, 178-, and 4-fold, respectively, in cells treated with rosiglitazone (Fig. 1a-c), and hereafter referred to as beige adipocytes. In comparison, markers associated with white adipocytes, Serpina3k [12], Wdnm1-like [8], and ASC1 [9] were greater in the cells that were not treated with rosiglitazone (Fig. 1d-f), and hereafter referred to as white adipocytes. With the notable exception of TMEM26, putative beige markers were elevated in beige adipocytes (Fig. 2a-h). However, only CAR4, FGF21, CITED1, CD137, and P2RX5 displayed a more than 2-fold elevation with CAR4 exhibiting the greatest elevation (23-fold: Fig. 2e). Hence all markers but TMEM26 fulfil the criteria of being expressed at higher levels in beige vs white adipocytes (Table 3).

**Table 3:** Summary of beigeing criteria met by the thermoregulatory genes and putative beige markers when studied under *in vitro* and *in vivo* conditions

Condition	UCP1	CIDEA	Cox8b	FGF21	P2RX5	PAT2	CITED1	CAR4	TBX1	CD137	TMEM26
(A) Beige > white cell culture*	✓	✓	✓	✓	✓	✓	✓	✓	✓	✓	
(B) Beige > white cell culture (adrenergic stimulation)	✓	✓	✓	✓	✓	✓		✓	✓	✓	
(C) Least changed by adrenergic stimulation cell culture		✓						✓			
(D) Elevated in WAT by cold exposure* (young adult mice)	✓	✓	✓	✓	✓	✓		✓			
(E) Large dynamic range in WAT* (young adult mice)	✓	✓	✓	✓							
(F) Elevated in WAT by cold exposure* (older mice)	✓	✓	✓								
(G) Adipocyte fraction > SVF*		✓	✓	✓	✓	✓					
(H) Elevated in adipocyte fraction by cold exposure*	✓	✓	✓	✓	✓	✓	✓	✓	✓		
(I) Beige adipocyte > SVF cells cell culture	✓	✓	✓	✓	✓	✓	✓	✓		✓	

A check mark indicates whether the thermoregulatory genes (columns 2 to 4) or beige transcripts (columns 5 to 12) denoted across the top row met the conditions of a beigeing marker detailed in the first column. The conditions are: A) the marker is significantly elevated in beige over white adipocytes in cell culture, B) the marker is significantly elevated in beige over white adipocytes in cell culture in the presence of adrenergic stimulation, C) the marker was least changed by adrenergic stimulation in beige and white adipocytes in cell culture, D) the marker was significantly elevated by cold exposure in the subcutaneous adipose tissue of young adult mice, E) the marker displayed at least a 2 fold dynamic range in the subcutaneous adipose tissue of young adult mice exposed to cold, F) the marker was significantly elevated by cold exposure in the subcutaneous adipose tissue of older mice, G) the marker was expressed at a significantly higher level in the adipocyte fraction compared to the stromal vascular fraction of subcutaneous adipose tissue of older mice reared at room temperature, H) the marker was significantly elevated by cold exposure in the adipocyte fraction of subcutaneous adipose tissue of older mice exposed to the cold, and I) the marker was expressed at significantly higher levels in SVF cells differentiated into beige adipocytes compared to undifferentiated SVF cells in culture. Conditions marked with an asterisk (*) denote criteria defined *a priori* to be met for a beigeing marker. The remaining conditions, B, C, and I were added based either on results employing adrenergic stimulation in cultured cells or differential expression of the markers in cultured SVF cells and beige adipocytes

### Beige adipocyte marker gene expression in white and beige adipocytes after adrenergic stimulation

Adrenergic stimulation increased UCP1 mRNA expression in both white and beige adipocytes with 11-fold greater expression in the beige compared to white adipocytes (Fig. 1a). Adrenergic stimulation reduced levels of white adipocyte markers as well as those of the transcript for Cox8b (Fig. 1c) in both white and beige cells. CIDEA mRNA levels did not change with adrenergic stimulation in both white and beige adipocytes (Fig. 1b). Adrenergic stimulation affected the levels of beige markers in complex ways in both white and beige cells. The levels of the transcript for P2RX5 and TBX1 where decreased in both cells types by stimulation (Fig. 2b and f), while those of PAT2 were decreased only in white adipocytes (Fig. 2c), and those of CAR4 and CD137 were increased only in white adipocytes (Fig. 2e and g). FGF21 was increased both in white and beige cells (Fig. 2a), but the levels were 8-fold greater in beige cells. Despite these changes the capacity to distinguish between beige and white adipocytes was maintained by all the markers except CITED1, which was increased in white adipocytes to such an extent that it lost the capacity to discriminate between both cell types Fig. 2d). Beige marker CAR4 was changed the least by adrenergic stimulation (Fig 2e). While not part of the *a priori* defined criteria for beige adipocyte markers, the responses of the different gene transcripts to adrenergic stimulation are summarized in Table 3.

### Age-dependent beige adipocyte marker gene expression in response to cold exposure

**Fig. 3:**
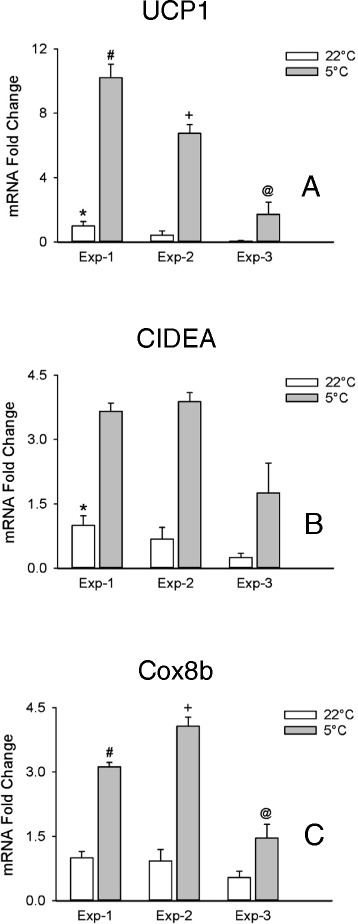
Transcripts of thermoregulatory genes in inguinal adipose tissue. The transcripts for UCP1 **a**, CIDEA **b**, and Cox8b (**c**) were evaluated in inguinal adipose tissue from mice maintained at room temperature (22 °C) or exposed to cold (5 °C) for one week in 3 separate experiments where the ages of the mice at the end of the experiments were 2 months and 10 days (Exp-1), 2 months and 23 days (Exp-2), and 4 months and 10 days (Exp-3). Transcripts were normalized to 22 °C in Experiment-1. Values are means ± SEM (*n* = 4–5). Comparisons of 22 °C vs 5 °C within each experiment for all transcripts were statistically significantly different (*P* ≤ 0.05) and are not marked with a symbol. (*) different from Exp-3, 22 °C, (#) different from Exp-2, 5 °C, (+) different from Exp-3, 5 °C, (@) different from Exp-1, 5 °C, *p* ≤ 0.05, two way ANOVA followed by multiple comparisons using the Holm-Sidak method

**Fig. 4:**
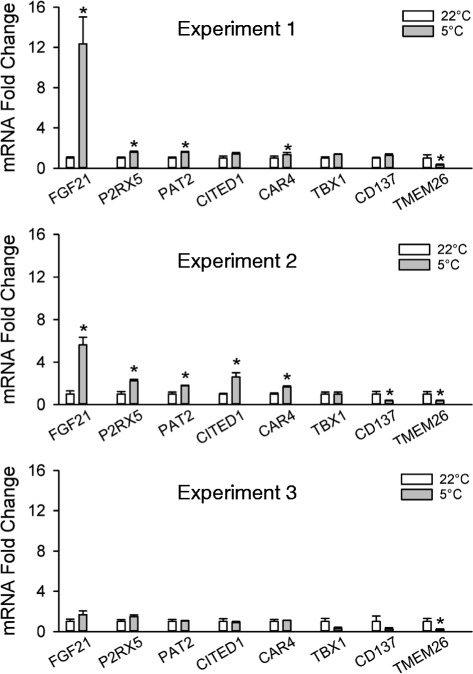
Markers of beige adipocytes in inguinal adipose tissue. The transcripts for FGF21, P2RX5, PAT2, CITED1, CAR4, TBX1, CD137, and TMEM26 were evaluated in inguinal adipose tissue from mice maintained at room temperature (22 °C) or exposed to cold (5 °C) for one week in 3 separate experiments were the ages of the mice at the end of the experiment were 2 months and 10 days (Exp-1), 2 months and 23 days (Exp-2), and 4 months and 10 days (Exp-3). The levels of the transcripts at 5 °C were normalized to the transcript of the same gene at 22 °C. Values are means ± SEM (*n* = 4–5). (*) different from 22 °C, *p* ≤ 0.05, two-tailed t-test

We employed mice 2 to 4 months of age to evaluate the possible effect of age on beigeing in WAT. Figures 3 and 4 show the result of 3 such experiments with mice that were 2 months and 10 days (experiment-1), 2 months and 23 days (experiment 2), or 4 months and 10 days (experiment 3) of age by the end of the experiment. In the 3 experiments the transcripts for UCP1, CIDEA, and Cox8b were significantly increased by cold treatment compared to room temperature (Fig. 3a-c). From the younger (Exp-1) to the older (Exp-3) mice there was a trend toward a decrease in the levels of the transcripts both in the mice kept at room temperature as well as those exposed to the cold, suggesting that the number of beige adipocytes decrease with age in mice. As shown in Fig. 4, the transcripts for FGF21, P2RX5, PAT2, and CAR4 were significantly elevated in the first two experiments. Of these, FGF21 displayed the greatest dynamic range, whereas the rest of the transcripts with the exception of P2RX5 and CITED1 in experiment 2 were elevated less than 2-fold, if at all. TBX1, CD137, and TMEM26 were not elevated by cold treatment in either experiment, and TMEM26 was significantly decreased in all 3 experiments. None of the beige markers including FGF21 were significantly increased over baseline in experiment 3 (Fig. 4) which employed older mice.

Therefore only the thermoregulatory gene transcripts meet the criteria that a beige marker should be increased in WAT in response to a beigeing stimulus and display a sufficiently high dynamic range in the mice of different ages evaluated here, whereas a distinction has to be made between young adult mice and older mice in the case of the proposed beige markers (Table 3).

We attempted to detect by Western blot UCP1, P2RX5, and FGF21 because they were among the markers that were consistently increased in the younger mice in response to cold treatment, and CD137 because of the disparate results we obtained *in vitro* vs *in vivo*. As expected, a large increase in UCP1 was detected in the inguinal adipose tissue of the mice exposed to the cold; however, the levels of the proteins for FGF21, P2RX5, and CD137 were below the limits of detection (see Additional file 1). Ussar et al., 2014, had previously reported that P2RX5 was undetectable in the inguinal adipose tissue of mice [9].

### Beige adipocyte marker gene expression in SVF cells and adipocytes

We separated the SVF from the adipocyte fraction of the 4 month and 10 day old mice from Experiment-3 (Figs. 3 and 4) to gauge whether beigeing in white adipose tissue can be better evaluated employing the isolated adipocyte fraction, and to determine the extent of the expression of the markers in both fractions and how it is changed by cold treatment. Transcripts for the markers of white adipocytes ASC1 and Wdnm1-like were 135- and 78-fold greater, respectively, in the adipocyte fraction, whereas the transcript for PDGFR-α, a marker for bipotential adipocyte precursors [13], was 25-fold greater in the SVF (see Additional file 2). This indicates that, at least in terms of the adipocytes and some of their precursors, the two fractions were successfully separated with minimal cross-contamination.

**Fig. 5:**
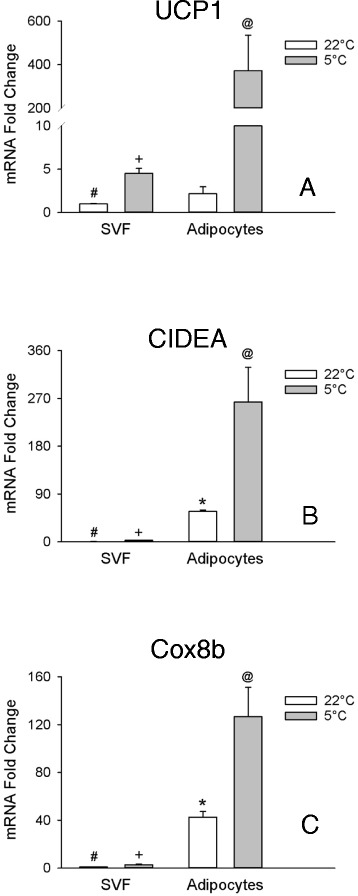
Transcripts of thermoregulatory genes in the adipocyte and SVF derived from inguinal adipose tissue. The transcripts for UCP1 **a**, CIDEA **b**, and Cox8b (**c**) were evaluated in the adipocyte and SVF of the 4 month and 10 day old mice from Experiment-3 (Figs. 3 and 4) kept at room temperature (22 °C) or exposed to the cold (5 °C) for one week. Transcripts were normalized to 22 °C, SVF. Values are means ± SEM (*n* = 3–5). (*) different from SVF 22 °C, (#) different from SVF 5 °C, (+) different from adipocytes 5 °C, (@) different from adipocytes 22 °C, *p* ≤ 0.05, two way ANOVA followed by multiple comparisons using the Holm-Sidak method

**Fig. 6:**
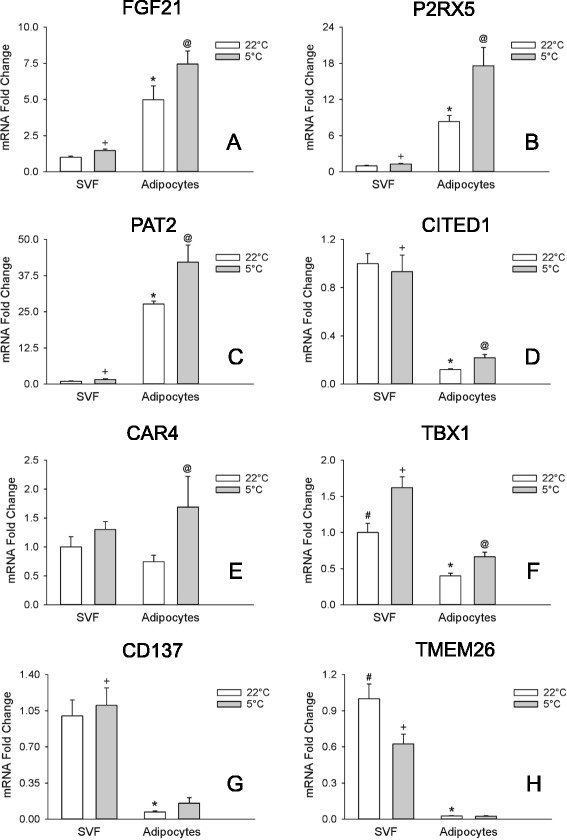
Markers of beige adipocytes in the adipocyte and SVF derived from inguinal adipose tissue. The transcripts for FGF21 **a**, P2RX5 **b**, PAT2 **c**, CITED1 **d**, CAR4 **e**, TBX1 **f**, CD137 **g**, and TMEM26 (**h**) were evaluated in the adipocyte and SVF of 4 month old mice kept at room temperature (22 °C) or exposed to the cold (5 °C) for one week. Transcripts were normalized to 22 °C, SVF. Values are means ± SEM (*n* = 3–5). (*) different from SVF 22 °C, (#) different from SVF 5 °C, (+) different from adipocytes 5 °C, (@) different from adipocytes 22 °C, *p* ≤ 0.05, two way ANOVA followed by multiple comparisons using the Holm-Sidak method

The transcripts for CIDEA and Cox8b, but not UCP1, were expressed at greater abundance in the adipocyte fraction compared to the SVF of mice housed at room temperature (Fig. 5a-c). Of all the beige markers tested only FGF21, P2RX5, and PAT2 (Fig. 6a-c) were expressed at greater levels (5-, 8-, and 28-fold greater, respectively) in the adipocyte fraction compared to the SVF from mice housed at room temperature. Levels of CAR4 did not differ between fractions (Fig. 6e). However TBX1 (Fig. 6f), CITED1 (Fig. 6d), and CD137 and TMEM26 (Fig. 6g-h) were expressed at lower levels (2.5-, 8-, 14-, and 33-fold lower, respectively) in the adipocyte fraction compared to the SVF in mice housed at room temperature. So only 2 thermoregularoty genes (CIDEA and Cox8b), and 3 beige markers (FGF21, P2RX5, and PAT2), meet the criteria of being expressed at a higher level in the adipocyte fraction of mice at room temperature (Table 3).

### Beige adipocyte marker gene expression in SVF cells and adipocytes in Response to Cold Exposure

The transcript for UCP1 was upregulated (173-fold) by cold stimulation in the adipocyte fraction, and the transcripts for CIDEA, and Cox8b were also increased by cold exposure, although to a lesser extent (Fig. 5). The beige markers CAR4 (Fig. 6e), P2RX5 (Fig. 6b), CITED1 (Fig. 6d), TBX1 (Fig. 6f), PAT2 (Fig. 6c), and FGF21 (Fig. 6a) were increased (128, 111, 83, 68, 52, and 50 %, respectively) in the adipocyte fraction by cold treatment. Only the transcripts for CD137 and TMEM26 (Fig. 6g-h) did not display a significant increase in the adipocyte fraction of those animals exposed to cold treatment. The net change in the levels of the transcripts in the SVF were small for most markers except for TBX1 where expression was increased to an extent comparable to that in the adipocyte fraction (Fig. 6f), and TMEM26 where the expression was decreased (Fig. 6h). Thus all gene transcripts but those of CD137 and TMEM26 meet the criteria of increased expression in the adipocyte fraction from subcutaneous WAT in response to a beigeing stimulus (Table 3).

### Beige adipocyte marker gene expression in cells from the SVF and beige adipocytes in culture

**Fig. 7:**
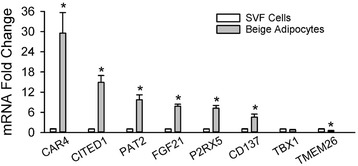
Beige markers in preadipocytes and SVF cells *in vitro*. The transcripts for CAR4, CITED1, PAT2, FGF21, P2RX5, CD137, TBX1, and TMEM26 were evaluated in SVF cells and beige adipocytes in culture. Transcripts were normalized to SVF cells. Values are means ± SEM (*n* = 3). (*) different from SVF, *p* ≤ 0.05, two-tailed t-test

The results obtained with the differential expression of the beige markers in the SVF and the adipocyte fraction led us to perform an additional experiment to evaluate the expression of beige markers *in vitro* in beige adipocytes and the cells from the SVF. Differentiation of cultured SVF cells into beige adipocytes should result in an increase in the levels of beige adipocyte markers. As expected the abundance of the transcripts for CIDEA, Cox8b, and UCP1 was much higher in beige adipocytes compared to the undifferentiated SVF cells (see Additional file 3). As shown in Fig. 7, the abundance of the transcripts of most beige adipocyte markers, including CD137, was significantly increased in beige adipocytes over that of undifferentiated SVF cells with the exception of TBX1, which did not increase, or TMEM26, which was reduced. While not part of the *a priori* defined criteria for beige adipocyte markers, these results are summarized in Table 3.

## Discussion

In this work we evaluated the expression of the transcripts for thermoregulatory genes and putative beige markers in cultured murine SVF cells differentiated into white and beige adipocytes, and in whole subcutaneous (inguinal) white adipose tissue, and its two major fractions (adipocyte fraction and SVF) of mice exposed to cold temperature to induce beigeing. Each of these approaches by itself has shortcomings. *C*ell culture conditions may generate results that differ from *in vivo* models. The results obtained from whole tissue represent the sum of the contributions of all cell types in the tissue. And the processing and time involved in the separation of the adipocytes and SVF may introduce changes to the relative levels of transcripts present in the cells *in vivo*. However, by characterizing and comparing the changes in gene expression obtained when employing each approach we can determine the best markers to evaluate beigeing.

TMEM26, CD137, and TBX1 were identified as beige adipocyte markers by Wu and coworkers [7], but other labs have had mixed success in validating these markers [9, 10]. Our data indicate that TBX1 may have some utility as a marker to discriminate beige and white adipocytes *in vitro*, or if the assay is performed on the adipocyte fraction from white adipose tissue. On the other hand, TMEM26 did not meet any of the criteria defined a priori or otherwise for a beige marker, and CD137 did not meet the criteria of being increased by a beigeing stimulus *in vivo*, but it seems to be an acceptable marker for beige adipocytes *in vitro*. One possibility to explain the limitations of TMEM26 and CD137 as beige markers is that suggested by Lee and coworkers, 2015. These authors found that the levels of CD137 and TMEM26 in cultured adipocyte precursors are decreased when differentiated into beige adipocytes [14]. The authors suggest that CD137 and TMEM26 may work better to identify beige adipocyte precursors rather than differentiated beige adipocytes in white adipose tissue. In agreement with these authors we found that TMEM26 was decreased when cultured cells from the SVF were differentiated into beige adipocytes. Therefore TMEM26 could be expressed at a higher abundance in the precursors of beige adipocytes in the SVF, and its levels decreased by the stimuli that produce beige adipocytes.

However, unlike the results of Lee and coworkers, 2015, we found that the expression of CD137 was increased when cultured cells from the SVF were differentiated into beige adipocytes, which is what would be expected of a beige adipocyte marker. Additional explanations as for why the use of CD137 may be challenging as a beige marker *in vivo* is that the transcript of this gene is expressed at a 10 fold higher abundance in the cells of the SVF and is regulated by other stimuli besides beigeing. CD137 is a member of the tumor necrosis factor (TNF) receptor superfamily of proteins which is expressed in immune cells like activated T cells and macrophages. CD137 participates in proinflamatory processes [15] and its levels are modified by inflammatory stimuli [16]. Thus, the large background signal due to the high expression of CD137 in the SVF, and its fluctuation in response to the inflammatory state of the tissue may make it difficult to evaluate the changes in the levels of the transcript exclusively due to beigeing.

FGF21, CAR4, and CITED1 were identified as beige adipocyte markers by microarray analyses of SVF cells induced to differentiate into brown, beige, or white adipocytes [8], and P2Rx5 and PAT2 were identified as brown/beige adipocyte markers employing a combination of *in silico*, *in vitro*, and *in vivo* methods [9]. We found that these markers can all be used to discriminate beige from white adipocytes *in vitro*. We also found that, with the exception of CITED1, the specificity of these markers is maintained even in the presence of adrenergic stimulation. The fact that short-term adrenergic stimulation modified the levels of most transcripts even in cells fully differentiated into a beige phenotype suggests that fluctuations in the levels of these transcripts can occur in a way that is independent of beige cell numbers. In this sense CIDEA and CAR4 may be useful markers because their expression was least affected by short-term adrenergic stimulation in either beige or white cells. Even though adrenergic stimulation increased the levels of UCP1 in both beige and white cells, it decreased the levels of the transcript for Cox8b. This may represent a negative feedback mechanism involved in regulating the cell’s response to a strong adrenergic stimulus.

Our data also indicated that, at least in the younger mice, FGF21, P2RX5, PAT2, and CAR4 are also markers of beige adipocytes, although only FGF21 exhibited a large dynamic range. It is interesting that all the beige markers that were consistently elevated in the first two *in vivo* experiments in response to the cold (FGF21, P2RX5, PAT2, and CAR4) were also the markers that were either expressed at greater (FGF21, P2RX5, and PAT2) or the same (CAR4) level in the adipocyte fraction compared to the SVF of animals kept at room temperature. In contrast the markers that either displayed no consistent increase (CITED1: increased in experiment 2, but not experiment 1) or no increase at all (TBX1, CD137, and TMEM26) in these two experiments were all expressed in the SVF at a greater abundance. A greater expression of a marker in the cells comprising the SVF may create a higher background that can make it harder for increases in expression of a beige marker in the adipocyte fraction to be detected in the intact tissue.

In experiment 3 performed with older animals the data were more variable than in experiments 1 or 2, and although the thermogenic markers UCP1, CIDEA, and Cox8b were significantly elevated by cold treatment, no significant elevations of beige markers were detected. The comparative evaluation of the expression of the transcripts for UCP1, CIDEA, and Cox8b among these experiments suggested that the extent of beigeing in the older mice was lower compared to younger mice, which would explain the decrease in the beige markers. A loss of UCP1 and multilocular adipocytes in subcutaneous adipose tissue with age has been reported in mice [17], so it is also possible that the capacity for beigeing in WAT of older mice in response to a stimulus such as cold exposure is reduced. This indicates that the use of beige markers in older mice may have limited validity. However, when we evaluated the markers in the adipocyte fractions of older mice, we were able to detect significant elevations of most markers in response to cold treatment with the exception of CD137 and TMEM26. So, evaluating the markers in the isolated adipocyte fraction may be one way to overcome this limitation.

Species-dependent difference, age differences, and animal housing temperature may contribute to differences in beige adipocyte marker gene expression across published studies. In a recent study to validate markers of adipose tissue depots by de Jong and coworkers [11], the authors used NMRI mice which are less prone to diet-induced obesity compared to the strain we used, C57BL/6 [18], and they exposed their mice to thermoneutral (30 °C) or cold temperatures (5 °C) for 3 weeks (compared to 22 °C and 5 °C for one week in our study). Despite these differences, the authors also found that in inguinal adipose tissue TBX1, CD137, and TMEM26 were not increased by cold treatment (they were significantly decreased) while FGF21, P2RX5, and PAT2 were increased with FGF21 having the greatest dynamic range. Although our current work has further contributed by highlighting the possible limitations of using beige markers (the age of the animals) as well as their differential expression among the adipocyte and SVFs, the agreement between our data and that of de Jong and coworkers indicate that our results are not specific to one strain of mice and one set of conditions.

While not technically markers of beige adipocytes, the transcripts of thermoregulatory genes like UCP1, CIDEA, and Cox8b can also be employed to evaluate beigeing in mouse WAT. We found that UCP1, CIDEA, and Cox8b fulfilled all of the criteria we defined for beige markers. Because of this and the fact that the stimulus we employed, cold exposure, is a powerful stimulus to induce beigeing, these thermoregulatory genes are likely better markers than the specific beige markers in studies where milder beigeing stimuli and/or older mice are employed. Nevertheless, the use of beige marker FGF21 may provide additional information about the dynamics of the beigeing process. FGF21 is a factor that induces beige cell differentiation in white adipose tissue, and its upregulation in the tissue may stimulate beige adipocyte differentiation through autocrine or paracrine effects [19].

Finally we must consider the applicability of our findings to humans. Our study clearly shows that even in the mouse the effectiveness of some beige markers may vary depending on several variables (*in vitro* vs *in vivo*, adrenergic stimulation, whole tissue vs fractions, age, etc.). Thus the results obtained from the application of beige markers validated in rodents to human samples should be interpreted with caution. There is a need for studies that evaluate these markers in human tissues exposed to beigeing stimuli. For example Kern and coworkers, 2014, found that in human subjects the thermoregulatory markers UCP1 and PGC1-α and the beige markers TMEM26 and TBX1 (but not CD137) were increased during winter in subcutaneous adipose tissue biopsies [20]. It is only through studies like these and the evaluation of in vitro differentiated cells and subcellular fractions in different physiological conditions that beige markers for humans will be fully validated.

## Conclusions

Researchers seeking to discriminate between cultured white and beige adipocytes or studying their possible interconversion can use any of the thermoregulatory genes or beige markers described herein with the exception of TMEM26. Researchers including adrenergic stimulation paradigms in their cell culture methodology should consider how even short-term stimulation can change the levels of the markers and note that CIDEA and CAR4 were the genes whose expression was least affected, whereas CITED1 lost its capacity to discriminate between beige and white adipocytes. Researchers seeking to evaluate the degree of beigeing in whole WAT of mouse models in response to experimental manipulations can employ UCP1, CIDEA, or Cox8b. The best beige markers for this purpose are FGF21, P2RX5, PAT2, or CAR4, but the low dynamic range of some of these markers, the age of the mice, and the strength of the beigeing stimulus may represent a limitation, which may be overcome by their evaluation in the adipocyte fraction.

## Additional files

Additional file 1: Western blots of UCP1, beta-actin, FGF21, P2RX5, and CD137 in WAT of control mice (22 °C) and mice exposed to cold (5 °C). (PPTX 332 kb) Additional file 2: Distribution of markers of white adipocytes and preadipocytes between the adipocyte fraction and SVF. The transcripts for ASC1 and Wdnm1-like (A) or PDGFR-alpha (B) were evaluated in the adipocyte fraction and SVF of 4 month and 10 days old mice kept at room temperature (22 °C). Transcripts were normalized to SVF. Values are means ± SEM (*n* = 3–5). (*) different from SVF, p ≤ 0.05, two-tailed t-test. (PPTX 97 kb) Additional file 3: Thermoregulatory markers in cells from the SVF and beige adipocytes *in vitro*. The transcripts for CIDEA, Cox8b, and UCP1 were evaluated in SVF cells and beige adipocytes in culture. Transcripts were normalized to SVF cells. Values are means ± SEM (*n* = 3). (*) different from SVF cells, p ≤ 0.05, two-tailed t-test. (PPTX 71 kb)

## Abbreviations

IBMX: isobutylmethylxanthine
Iso: isoproterenol
SVF: stromal vascular fraction
WAT: white adipose tissue

## References

[1] Merlin J, Evans BA, Dehvari N, Sato M, Bengtsson T, Hutchinson DS (2015). Could burning fat start with a brite spark? Pharmacological and nutritional ways to promote thermogenesis. Mol Nutr Food Res.

[2] Feldmann HM, Golozoubova V, Cannon B, Nedergaard J (2009). UCP1 ablation induces obesity and abolishes diet-induced thermogenesis in mice exempt from thermal stress by living at thermoneutrality. Cell Metab.

[3] Harms M, Seale P (2013). Brown and beige fat: development, function and therapeutic potential. Nat Med.

[4] Schulz TJ, Huang P, Huang TL, Xue R, McDougall LE, Townsend KL (2013). Brown-fat paucity due to impaired BMP signalling induces compensatory browning of white fat. Nature.

[5] Cohen P, Levy JD, Zhang Y, Frontini A, Kolodin DP, Svensson KJ (2014). Ablation of PRDM16 and beige adipose causes metabolic dysfunction and a subcutaneous to visceral fat switch. Cell.

[6] Stanford KI, Middelbeek RJ, Townsend KL, Lee MY, Takahashi H, So K (2015). A novel role for subcutaneous adipose tissue in exercise-induced improvements in glucose homeostasis. Diabetes.

[7] Wu J, Bostrom P, Sparks LM, Ye L, Choi JH, Giang AH (2012). Beige adipocytes are a distinct type of thermogenic fat cell in mouse and human. Cell.

[8] Sharp LZ, Shinoda K, Ohno H, Scheel DW, Tomoda E, Ruiz L (2012). Human BAT possesses molecular signatures that resemble beige/brite cells. PLoS One.

[9] Ussar S, Lee KY, Dankel SN, Boucher J, Haering MF, Kleinridders A (2014). ASC-1, PAT2, and P2RX5 are cell surface markers for white, beige, and brown adipocytes. Sci Transl Med.

[10] Rosenwald M, Perdikari A, Rulicke T, Wolfrum C (2013). Bi-directional interconversion of brite and white adipocytes. Nat Cell Biol.

[11] de Jong JM, Larsson O, Cannon B, Nedergaard J (2015). A stringent validation of mouse adipose tissue identity markers. Am J Physiol Endocrinol Metab.

[12] Kajimura S, Seale P, Tomaru T, Erdjument-Bromage H, Cooper MP, Ruas JL (2008). Regulation of the brown and white fat gene programs through a PRDM16/CtBP transcriptional complex. Genes Dev.

[13] Lee YH, Petkova AP, Mottillo EP, Granneman JG (2012). In vivo identification of bipotential adipocyte progenitors recruited by beta3-adrenoceptor activation and high-fat feeding. Cell Metab.

[14] Lee MW, Odegaard JI, Mukundan L, Qiu Y, Molofsky AB, Nussbaum JC (2015). Activated type 2 innate lymphoid cells regulate beige fat biogenesis. Cell.

[15] Kim CS, Kim JG, Lee BJ, Choi MS, Choi HS, Kawada T (2011). Deficiency for costimulatory receptor 4-1BB protects against obesity-induced inflammation and metabolic disorders. Diabetes.

[16] Tu TH, Kim CS, Goto T, Kawada T, Kim BS, Yu R (2012). 4-1BB/4-1BBL interaction promotes obesity-induced adipose inflammation by triggering bidirectional inflammatory signaling in adipocytes/macrophages. Mediators Inflamm.

[17] Rogers NH, Landa A, Park S, Smith RG (2012). Aging leads to a programmed loss of brown adipocytes in murine subcutaneous white adipose tissue. Aging Cell.

[18] Matyskova R, Maletinska L, Maixnerova J, Pirnik Z, Kiss A, Zelezna B (2008). Comparison of the obesity phenotypes related to monosodium glutamate effect on arcuate nucleus and/or the high fat diet feeding in C57BL/6 and NMRI mice. Physiol Res.

[19] Fisher FM, Kleiner S, Douris N, Fox EC, Mepani RJ, Verdeguer F (2012). FGF21 regulates PGC-1alpha and browning of white adipose tissues in adaptive thermogenesis. Genes Dev.

[20] Kern PA, Finlin BS, Zhu B, Rasouli N, McGehee RE, Westgate PM (2014). The effects of temperature and seasons on subcutaneous white adipose tissue in humans: evidence for thermogenic gene induction. J Clin Endocrinol Metab.

