# Supplementary figures and images for: Evaluation of markers of beige adipocytes in white adipose tissue of the mouse
Source: Nutr Metab (Lond). 2016 Mar 18;13:24. doi: 10.1186/s12986-016-0081-2 (PMC4797138; doi:10.1186/s12986-016-0081-2)

## Slide 1
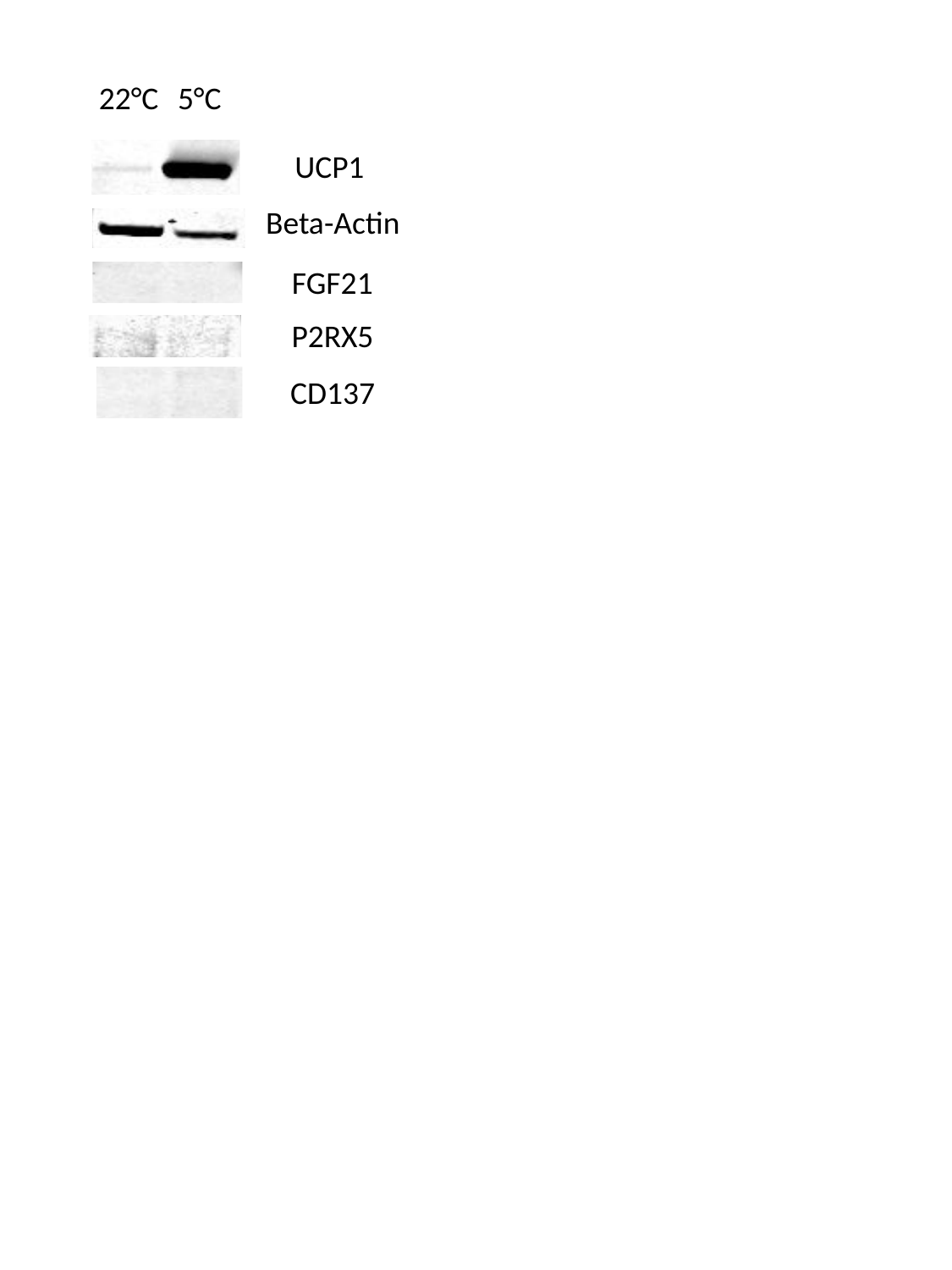

22°C
5°C
UCP1
Beta-Actin
FGF21
P2RX5
CD137

Supplement: Additional file 1: — Western blots of UCP1, beta-actin, FGF21, P2RX5, and CD137 in WAT of control mice (22 °C) and mice exposed to cold (5 °C). (PPTX 332 kb) [file 12986_2016_81_MOESM1_ESM.pptx]

## Slide 1
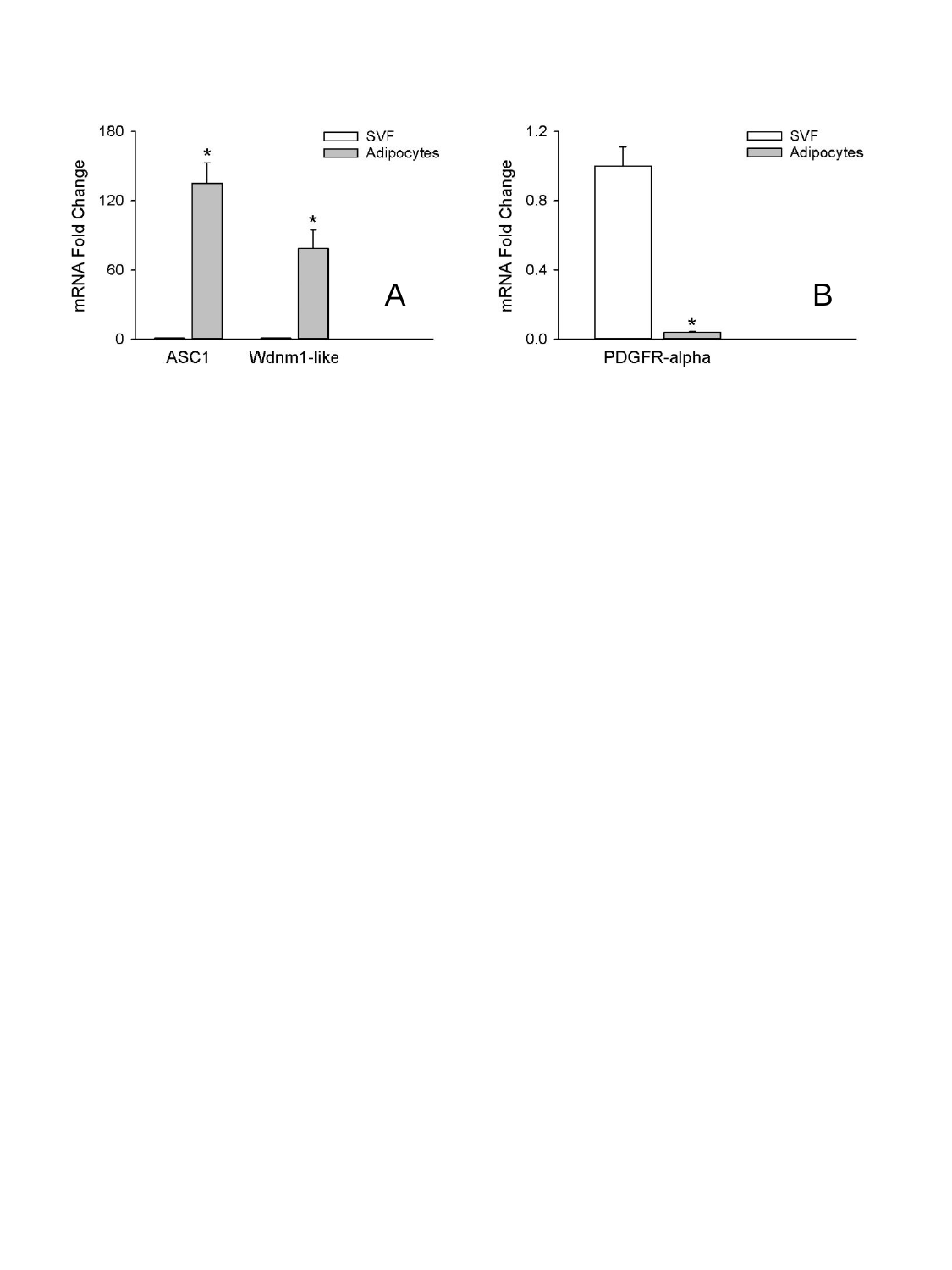

A
B

Supplement: Additional file 2: — Distribution of markers of white adipocytes and preadipocytes between the adipocyte fraction and SVF. The transcripts for ASC1 and Wdnm1-like (A) or PDGFR-alpha (B) were evaluated in the adipocyte fraction and SVF of 4 month and 10 days old mice kept at room temperature (22 °C). Transcripts were normalized to SVF. Values are means ± SEM (n = 3–5). (*) different from SVF, p ≤ 0.05, two-tailed t-test. (PPTX 97 kb) [file 12986_2016_81_MOESM2_ESM.pptx]

## Slide 1
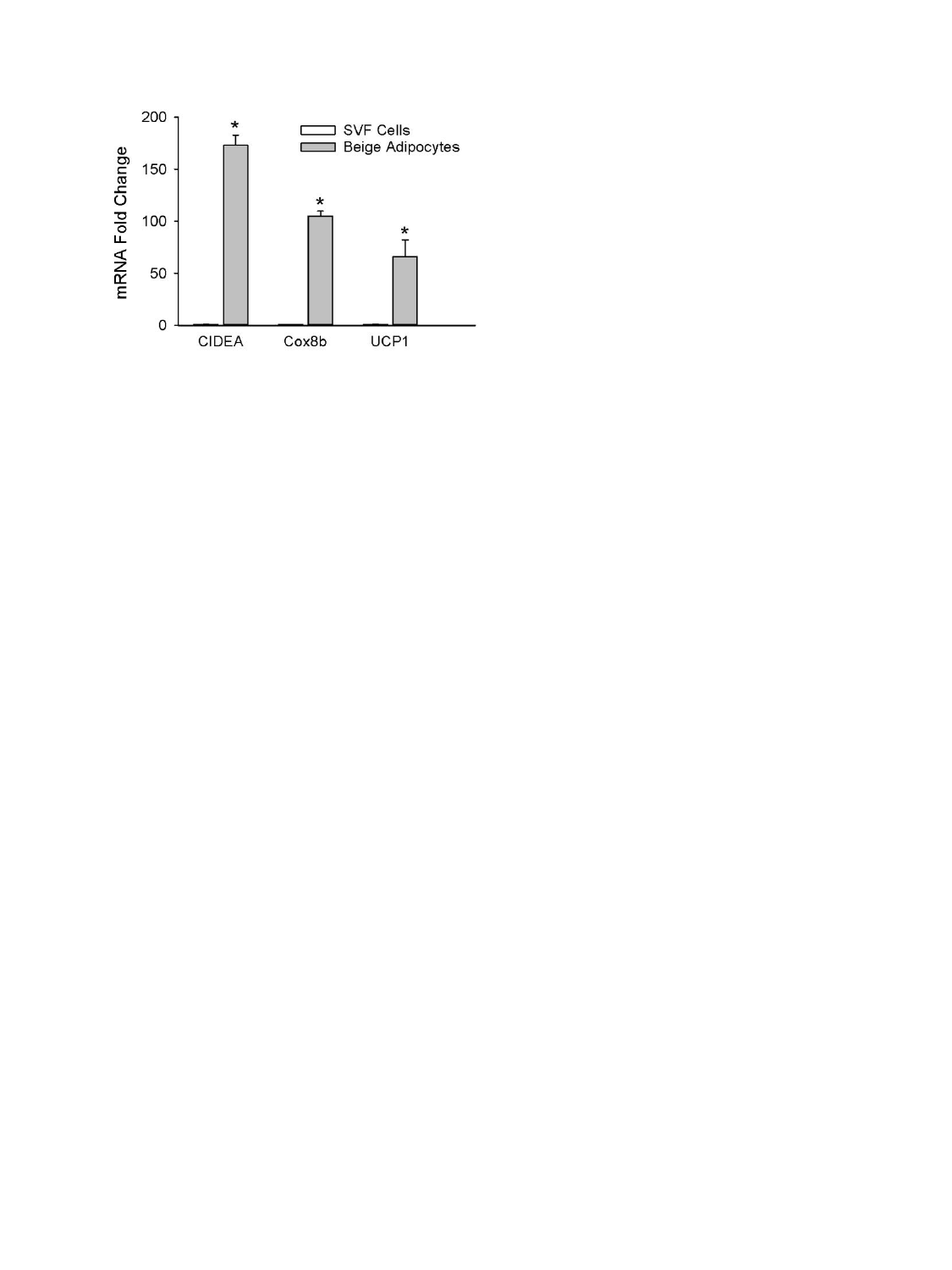

Supplement: Additional file 3: — Thermoregulatory markers in cells from the SVF and beige adipocytes in vitro. The transcripts for CIDEA, Cox8b, and UCP1 were evaluated in SVF cells and beige adipocytes in culture. Transcripts were normalized to SVF cells. Values are means ± SEM (n = 3). (*) different from SVF cells, p ≤ 0.05, two-tailed t-test. (PPTX 71 kb) [file 12986_2016_81_MOESM3_ESM.pptx]
